# Mutational Pressure in Zika Virus: Local ADAR-Editing Areas Associated with Pauses in Translation and Replication

**DOI:** 10.3389/fcimb.2017.00044

**Published:** 2017-02-22

**Authors:** Vladislav V. Khrustalev, Tatyana A. Khrustaleva, Nitin Sharma, Rajanish Giri

**Affiliations:** ^1^Department of General Chemistry, Belarusian State Medical UniversityMinsk, Belarus; ^2^Laboratory of Cellular Technologies, Institute of Physiology of the National Academy of Sciences of BelarusMinsk, Belarus; ^3^School of Basic Sciences, Indian Institute of Technology MandiMandi, India

**Keywords:** Zika virus, mutational pressure, pauses in replication, ADAR-editing, ribosome stalling

## Abstract

Zika virus (ZIKV) spread led to the recent medical health emergency of international concern. Understanding the variations in virus system is of utmost need. Using available complete sequences of ZIKV we estimated directions of mutational pressure along the length of consensus sequences of three lineages of the virus. Results showed that guanine usage is growing in ZIKV RNA plus strand due to adenine to guanine transitions, while adenine usage is growing due to cytosine to adenine transversions. Especially high levels of guanine have been found in two-fold degenerated sites of certain areas of RNA plus strand with high amount of secondary structure. The usage of cytosine in two-fold degenerated sites shows direct dependence on the amount of secondary structure in 52% (consensus sequence of East African ZIKV lineage)—32% (consensus sequence of epidemic strains) of the length of RNA minus strand. These facts are the evidences of ADAR-editing of both strands of ZIKV genome during pauses in replication. RNA plus strand can also be edited by ADAR during pauses in translation caused by the appearance of groups of rare codons. According to our results, RNA minus strand of epidemic ZIKV strain has lower number of points in which polymerase can be stalled (allowing ADAR-editing) compared to other strains. The data on preferable directions of mutational pressure in epidemic ZIKV strain is useful for future vaccine development and understanding the evolution of new strains.

## Introduction

Zika virus (ZIKV) is a pathogenic mosquito borne virus that became a Health emergency in February, 2016 (WHO, 2016) (http://www.who.int/mediacentre/news/statements/2016/emergency-committee-zika-microcephaly/en/). ZIKV is a member of flaviviridae family and transmitted by Aedes vector to cause severe neurological disorders such as fetal microcephaly and Guillain-Barré syndrome (Cao-Lormeau et al., 2016; Heymann et al., 2016). ZIKV infection has been known since 1940s, when the studies on yellow fever virus (YFV) yielded the isolation of Zika strain in Uganda (Wikan and Smith, 2016). But the expansion of geographic range of ZIKV has been observed after the significant outbreak infecting over 65% of population in Yap Island, Micronesia in 2007 (Duffy et al., 2009). The rapid spread of ZIKV during the outbreak in French Polynesia (2013), as well as the Brazilian outbreak in 2015 has increased the risk of infection worldwide. A surge in the number of fetal microcephaly cases in Brazil has gained the attention toward ZIKV infection (Butler, 2016). The evidences of ZIKV vertical transmission during pregnancy have raised alarms and posed a situation of global threat due to high epidemic and less effective control measures of infection (Coyne and Lazear, 2016). This results into the drastic increase in clinical and research framework to find effective strategies to encounter ZIKV infection (Giri et al., 2016).

ZIKV is a positive sense single stranded RNA virus with genomic size of ~10.7 kb. The genome of ZIKV encodes a single polyprotein that is cleaved into three structural and seven non-structural proteins. There are three major lineages of ZIKV: from West Africa, East Africa, and Asia. The Asian lineage has been evolved to give rise to modified strains detected in 2007 that are responsible for neurological complications (Haddow et al., 2012). The epidemiological evidences had revealed the transmission of ZIKV originated from Yap in 2007 to other Pacific islands, South, and Central America (Angeletti et al., 2016). The reference strain of ZIKV from GenBank belongs to the East African lineage (“type 1” in this study). West African lineage is referred to as “type 2” in this work, while Asian lineage (together with strains from the recent outbreak) goes under the name “epidemic strain,” or “type 3”.

Viral genomes are likely to show highest mutational frequencies and evolutionary evidences in living world. The fast mutation rate of RNA virus genomes creates different populations of viruses from a single culture termed as viral quasi-species. This kind of variation is thought to be reversible: the frequencies of different quasi-species vary during the infection process in the response to immune pressure and other factors. The pattern of mutations in viral genome is never random. Some types of nucleotide mutations always occur more frequently than other types. This situation is known under the name directional mutational pressure (Sueoka, 1993). Mutational pressure introduces irreversible changes in the nucleotide content of the whole viral population. For example, increased rates of A–G mutations will slowly make the usage of G higher in all the possible quasi-species. The causes of such unequal rates of mutations in RNA viruses may be different: error-prone polymerase, RNA editing, oxidative damage (Gros et al., 2002). One of the enzymes involved in viral RNA-editing is known as ADAR (double-stranded RNA-specific adenosine deaminase; Tomaselli et al., 2015). Expression of this enzyme is stimulated by the increase of the level of alien RNA in a cell (Tomaselli et al., 2015). However, some viruses provoke ADAR expression (including those from Flaviviridae family: Hepatitis C virus from hepacivirus genus and Bovine viral diarrhea virus from pestivirus genus), while others do not. Interestingly, just certain strains of Dengue virus promote ADAR expression (Umareddy et al., 2008). The direct consequence of RNA editing by ADAR is the increase of A–G transition rates (Tomaselli et al., 2015). So, one can suspect that genome of a given virus is edited by ADAR if the level of G in its RNA is high (Cuevas et al., 2016). High level of G in viral RNA increases the percentage of nucleotides forming doublestranded fragments. That is how ADAR-editing leads to rearrangements of secondary structure of RNA and the inclusion of previously unavailable adenine residues into doublestranded fragments.

It is important to state that the direction of mutational pressure may not be constant throughout the whole length of the viral genome (Khrustalev et al., 2015b). The same situation (local mutational pressures) has been found in certain bacterial and eukaryotic genes (Khrustalev et al., 2014, 2015a). Finding the cause of the deviation from the general mutational pattern may be even more useful procedure than estimating the general direction of mutational pressure. Autonomic transcription is thought to be the cause of local mutational pressure in bacteria and eukaryotic organisms (Khrustalev et al., 2014, 2015a). However, there is no autonomic transcription of small coding regions in Zika virus. In this article we described a hypothesis of translation-associated mutational pressure occurring in regions of RNA-plus strand situated after the sequences on which ribosome is stalled. We also hypothesize that RNA-dependent-RNA-polymerase stalling may open up the door to RNA-editing enzymes acting on fragments of RNA situated after such “stop-signals” as G-quarduplexes.

The knowledge on the main directions of mutational pressure should be used in vaccine design studies. One of the applications of this knowledge is in the choice of a best vector for DNA vaccine. The closer the pattern of mutations in the virus to the pattern of mutations in the vector, the higher the chance that vaccine vector will cover the spectrum of variants which occur during mutagenesis of wild type virus (Khrustalev et al., 2015b). Another application is in the determination of the less mutable fragments of coding regions. Such fragments should have the lowest amount of highly mutable nucleotides, especially, in first and second codon positions (Khrustalev et al., 2015b). Our findings have emphasized the importance of local mutational pressures analysis in understanding epidemics and disease tracking of ZIKV infection.

## Materials and methods

We used 79 complete sequences of Zika virus available in GenBank. All sequences have been aligned with MEGA 7.0 program using MUSCLE algorithm (Kumar et al., 2016). After that we deleted short sequences from 5′ and 3′-ends to let them start and end at the same position. So, actual first nucleotide in our sequences has number 218 in the reference Zika sequence (NC_012532.1), while the last nucleotide has number 10088 in that sequence. We analyzed only the coding region of the virus.

The phylogenetic analysis has shown three main clusters of Zika sequences (Tamura-Kumar method for evolutionary distances calculation, and minimum evolution method for the phylogenetic tree construction; Kumar et al., 2016). The first cluster (type) contains 13 sequences together with the reference one (East Africa lineage). The second cluster (type) contains nine sequences (West Africa lineage). The third cluster (Asian lineage) includes 55 sequences mostly from the recent outbreaks of the infection (2014–2016). There are also two sequences that occupy uncertain positions in the dendrogram (between two types of the virus), which we did not include in the following steps of the study. All sequences are available in the Supplementary Material File “Data Sheet 1.xlsx”

For each of the three types of virus we built a consensus sequence that has been analyzed with the help of “VVTAK Sliding Window,” QGRS Mapper, and RNAFold algorithms. Consensus sequences are available as Supplementary Material File “Data Sheet 3.xlsx” Nucleotide usages in four- and two-fold degenerated sites from third codon positions have been calculated by the “VVTAK Sliding Window” algorithm. We used the length of a sliding window equal to 150 codons (450 nucleotides) and the step of a sliding window equal to 1 codon. Positions of sequences able to form G-quarduplexes have been found by the QGRS Mapper (Kikin et al., 2006). Secondary structure of a single RNA strand (stems and loops) has been predicted using the RNAFold algorithm (we used centroid prediction; Hofacker and Stadler, 2006). The length of a sliding window for secondary structure prediction was equal to 400 nucleotides; the step was equal to 200 nucleotides. Reverse complement sequences have been created with the help of MEGA 7.0 program (Kumar et al., 2016). We executed QGRS and RNAFold predictions on reverse complement sequences as well.

Codon usage has been calculated in three consensus sequences with the help of MS Excel. Average codon usage in coding regions of *Homo sapiens, Pan troglodytes, Gorilla gorilla, Pongo pygmaeus*, and *Aedes aegypti* has been taken from the Codon Usage Database (Nakamura, 2000). There are just 10 codons with extremely low (<1%) usages in primates: CGU; CGA; UCG; UUA; CUA; AUA; GUA; CCG; ACG; GCG. We identified positions of sequences three codons in length in which at least two rare codons are situated throughout the length of each consensus sequence. In *Aedes aegypti* the number of rare codons is higher than in primates (there are 15 rare codons: UUA; CUU; CUA; AUA; GUA; UCU; UCA; CCU; ACA; UGU; CGA; CGG; AGA; AGG; GGG). Because of this the frequency of groups of rare codons usage is 5.375 times higher in the coding region of East Africa Zika lineage if we consider *Aedes aegypti* codon usage instead of *Homo sapiens* one. For West Africa Zika lineage this ratio is 6.021; for Asian Zika lineage it is equal to 5.680. So, rare codons for the *Aedes aegypti* are distributed relatively equally through the whole coding region of the viral genome, while rare codons for primates are distributed none equally: there are some long regions free from the groups of rare codons. So, we focused on the description of the distribution of rare codons for primates along the length of Zika coding region in the current study.

To confirm the existence of correlation between guanine usage in two-fold degenerated sites (G2f3p) on the plus strand of RNA and the percent of nucleotides in “stems” of the same strand we calculated the coefficient of correlation between G2f3p and the percent of stems. We calculated G2f3p usage in windows 150 codons in length and the percent of nucleotides in stems in windows 400 nucleotides in length. Five consequent pairs of these two windows (with centers at the same nucleotides) are referred to as a “dot.” So, the coefficient of correlation between G2f3p and the percent of stems has been calculated in each dot along the length of a nucleotide sequence. To find the area with a correlation between the percent of “stems” and the usage of guanine in two-fold degenerated sites we took into account the sequence of “dots” in which the correlation has been found interrupted by maximum three dots without such correlation. The same kind of analysis has been applied for guanine usage in four-fold degenerated sites (G4f) and the general guanine usage (G) with the secondary structure of the RNA plus strand; for cytosine usage in two-fold degenerated sites (C2f3p), in four-fold degenerated sites (C4f) and the general cytosine usage (C) with the secondary structure of the RNA minus strand.

The difference between nucleotide usage in two-fold degenerated sites in windows of 150 codons in length between consensus sequences of epidemic and reference Zika strands has been calculated with the help of MS Excel.

Directions of mutational pressure have been estimated using the “VVTAK VarInvar” algorithm in windows 400 codons in length with a step equal to 200 codons. This operation has been performed for the complete set of sequences from each of the three lineages. If the usage of a nucleotide is higher in invariable sites than in all stable sites (which stay two-fold degenerated or four-fold degenerated in all the sequences from the alignment), then the usage of such nucleotide is increasing (Sueoka, 1993). If the usage of a nucleotide is lower in invariable sites than in all stable sites, then the algorithm postulates that the usage of such nucleotide is decreasing (Sueoka, 1993). The algorithm takes into consideration just four-fold degenerated sites and two-fold degenerated sites from third codon positions. Results of such calculations are available as Supplementary Material File “Data Sheet 2.xlsx”

Variable two-fold degenerated sites have been found with the help of the “VVTAK VarInvar” algorithm in each of the three alignments of Zika sequences. Stable two-fold degenerated sites have been found with the same algorithm.

To check whether adenine in nucleotide sequences specific for ADAR2 editing mutates more frequently than in all positions, we calculated the usage of UAG, UAU, AAG, and AAU trinucleotides in three consensus sequences of Zika lineages and their reversed complements, and counted the number of mutated adenines in centers of these trinucleotides. The percent of mutated adenines in these ADAR2-specific motifs has been compared with the overall number of mutated adenines using two-tailed *t*-test.

## Results

### Nucleotide usage biases along the length of the consensus open reading frame of the East African Zika strains

Nucleotide usage in four-fold degenerated sites represents the direction of the most frequent types of nucleotide mutations. Since all the mutations in such sites are not leading to substitutions in amino acid sequence of a corresponding protein, they are considered to be neutral for the evolution of a protein. Of course, mutations in four-fold degenerated sites may influence the fate of the protein (by the way of the influence on RNA structure, on RNA interference, on the usage of more or less frequent codons, etc.), but not its primary structure (Cristina et al., 2016). So, the influence of natural selection on four-fold degenerated sites is lower than that on all other sites of a coding region.

**Figure 1:**
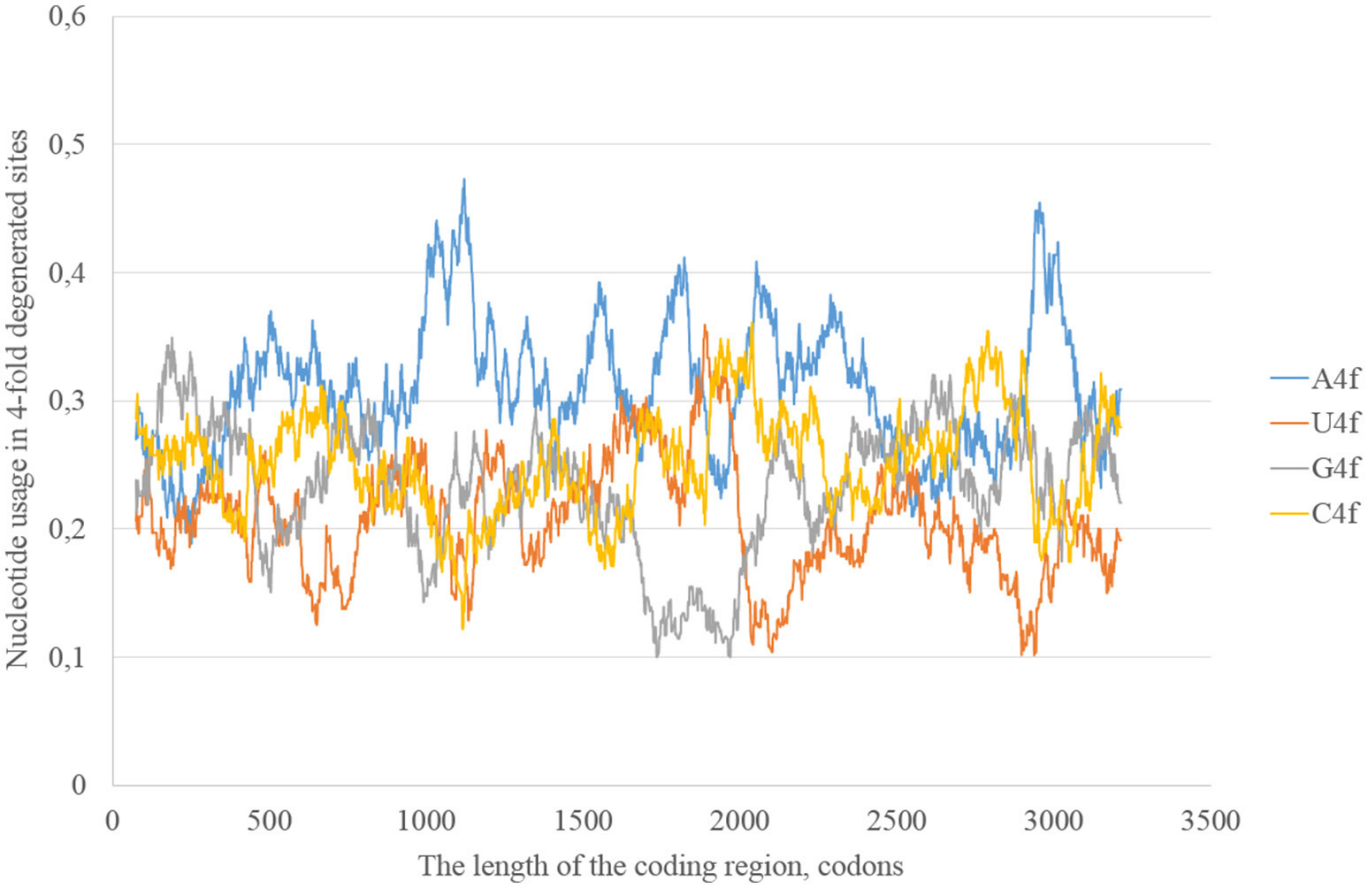
**Nucleotide usage in four-fold degenerated sites along the length of the consensus sequence for East African (type 1) ZIKV strains**.

In the consensus sequence of the reference Zika virus strain adenine usage prevails in four-fold degenerated sites (see Figure 1). However, its actual usage (A4f) is not something constant through the whole length of the sequence. In some sliding window (150 codons in length) A4f is higher than 40%, in others it is lower than 25%. In general, mutations of three other nucleotides to adenine occur at higher rates than mutations of adenine to three other nucleotides. Because of this, adenine usage is high in four-fold degenerated sites. In some fragments of ZIKV RNA, this type of mutation (from other nucleotides to adenine) is either occurring faster or fixing more easily producing the “waives” of A4f in the graph (Figure 1).

**Figure 2:**
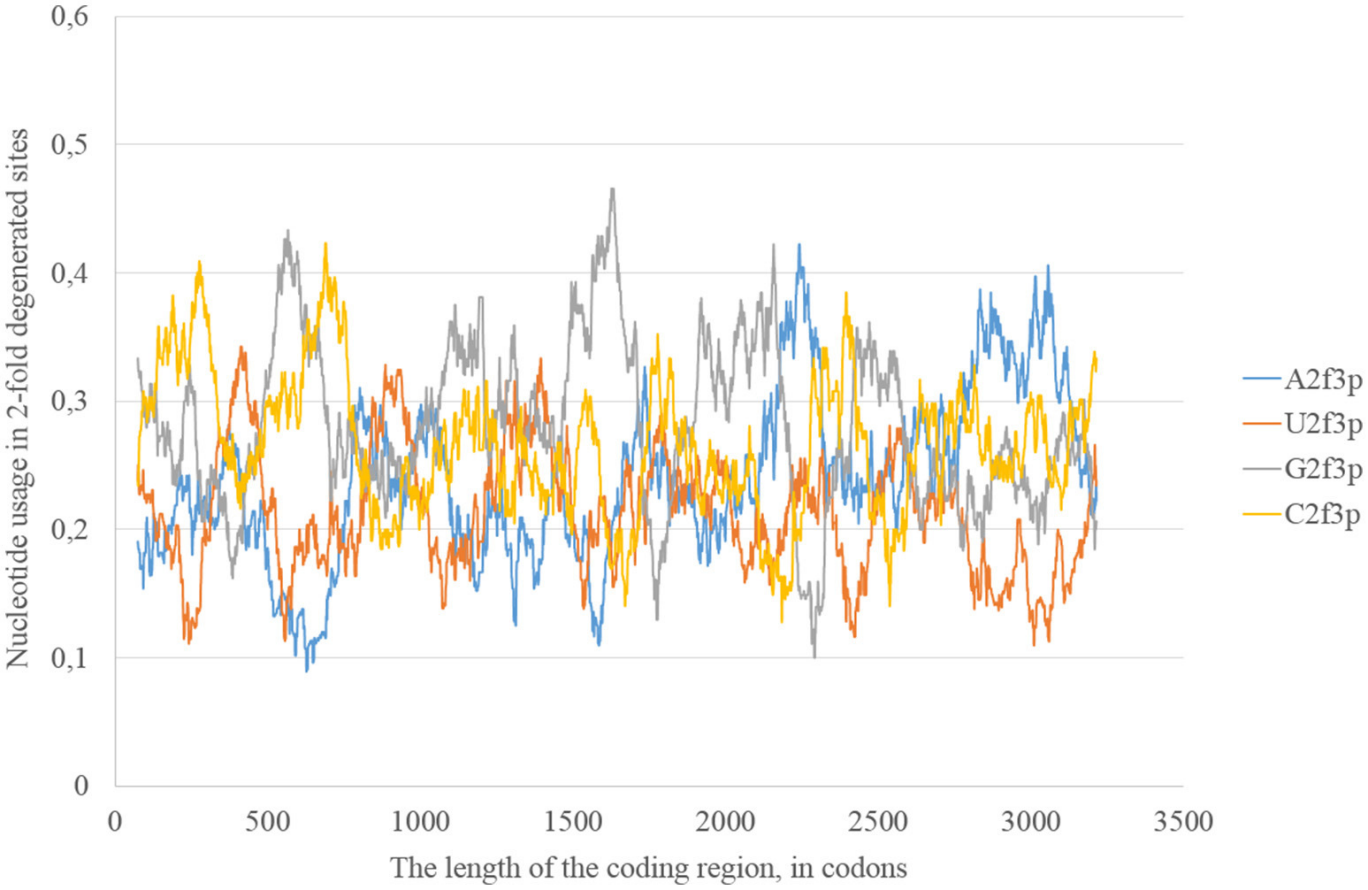
**Nucleotide usage in two-fold degenerated sites from third codon positions along the length of the consensus sequence for East African (type 1) ZIKV strains**.

The usage of nucleotides in two-fold degenerated sites from third codon positions represents the preferable direction of transitions. Indeed, just a transition (and not transversions) is neutral in two-fold degenerated sites from third codon positions. Transversions occurring in such sites lead to amino acid replacements. In the reference Zika strain guanine usage in two-fold degenerated sites (G2f3p) demonstrates several high peaks through the length of the coding region (Figure 2). Interestingly, the usage of adenine in two-fold degenerated sites is rather low. Only in two fragments closer to the 3′-end of the RNA plus strand the usage of A2f3p suddenly increases (Figure 2). The usage of cytosine in two-fold degenerated sites is also high in several fragments of the coding region.

According to the distribution of adenine usage in four- and two-fold degenerated sites, transversions leading to the growth of adenine usage are responsible of the high A4f levels. In contrast, transitions from G to A are less frequent then transitions from A to G in the most of the fragments of viral RNA plus strand.

The most probable mechanism of A–G transitions is the deamination of adenine leading to the formation of inosine that usually forms hydrogen bonds with cytosine (George et al., 2014). The process may be spontaneous or enzymatic. In the latter case enzymes from adenosine-RNA-deaminase (ADAR) family bind double-stranded (and not single-stranded) fragments of RNA and perform RNA-editing. So, Zika RNA plus strand may collect A to G transitions in both two- and four-fold degenerated sites, but then the level of G in four-fold degenerated sites decreases due to transversions (since G4f is lower than A4f).

The level of adenine should grow mostly due to transversions. The probable mechanism of its growth is a frequent oxidation of guanine on the RNA minus strand resulting in C to A transversions on the RNA plus strand. Such process can occur on the RNA plus strand as well. However, RNA minus strand may serve as a collector of mutations: multiple RNA plus strands are synthesized from the same RNA minus strand. Therefore, each next RNA plus strand should have more C to A transversions. If adenine deamination occurs on the RNA minus strand, then it should increase the usage of C2f3p on the RNA plus strand.

In general, we can observe that mutational pressure has two opposite directions in the same virus. Guanine usage is growing due to A to G transitions leading to the increase of G2f3p, and not G4f. Adenine usage is growing due to C to A transversions leading to the increase of A4f, and not A2f3p. Moreover, there are some areas in which G2f3p is much higher than in other areas.

### Secondary structure of RNA strands from East African Zika strains

**Figure 3:**
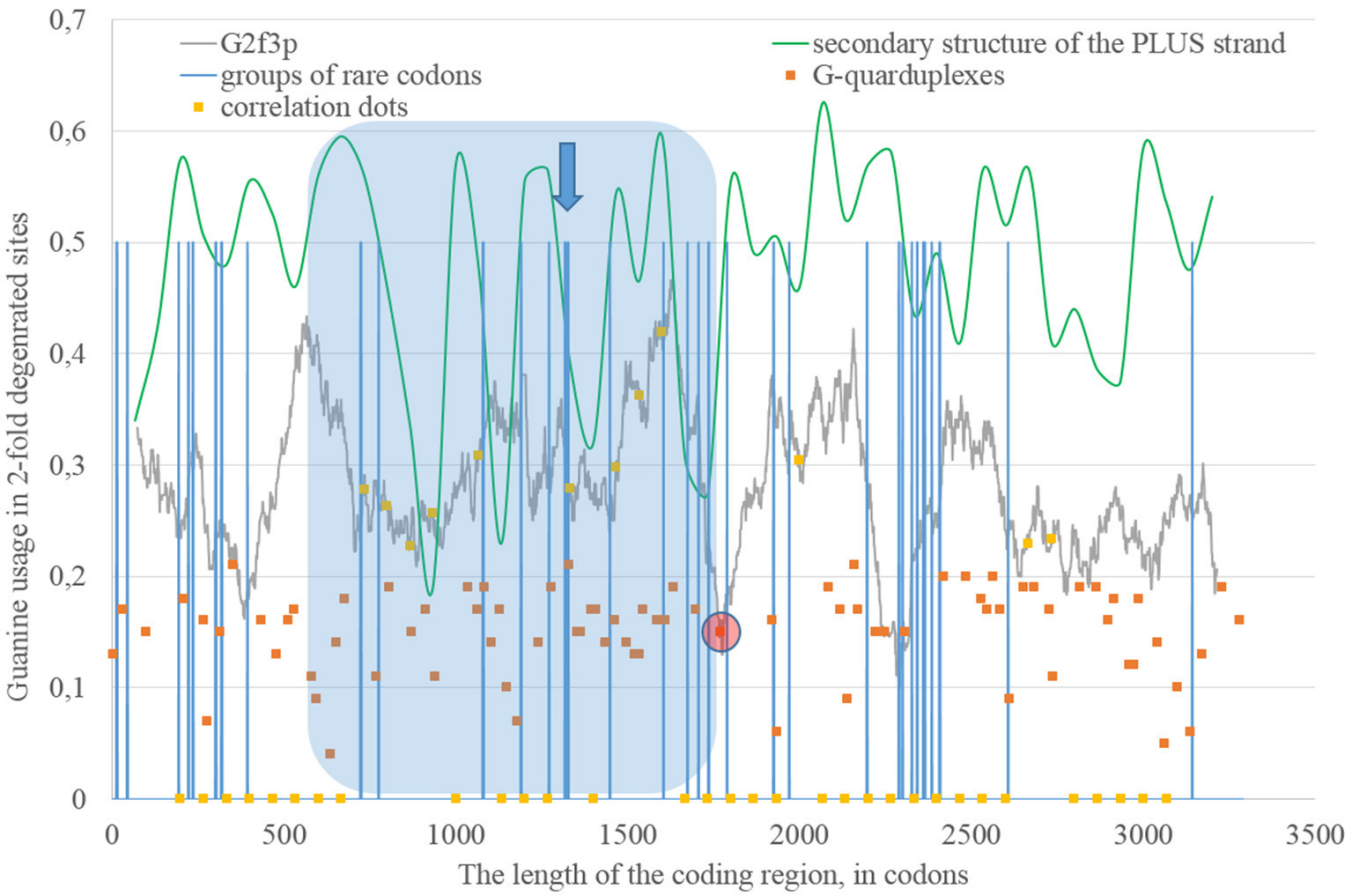
**The area of correlation (blue area) between the amount of nucleotides forming secondary structure of the RNA plus strand from ZIKV type 1 and the usage of guanine in two-fold degenerated sites from third codon positions (G2f3p)**. Positions of groups of rare codons and predicted G-quarduplexes are shown. If correlation dots are situated on the G2f3p line, then the coefficient of correlation between G2f3p and the percent of stems in five windows (150 codons and 400 nucleotides in length, respectively) is higher than 0.3.

Secondary structure of a single RNA strand forms because nucleotides make hydrogen bonds with each other. Perfect or imperfect inverted repeats usually make hairpins. Some of such hairpins play important roles in the viral lifecycle. For example, hairpins from 3′ or 5′-end of viral genome usually participate in replication (Thurner et al., 2004). Hairpins are also involved in the regulation of transcription and translation (Hofacker and Stadler, 2006). However, the most of the hairpins formed by RNA may not play any significant functional role. Obviously, they occur not just in untranslated, but in translated parts of RNA. Theoretically, “stems” of hairpins from Zika RNA plus strand should be prone to ADAR editing more than “loops” (single-stranded fragments). If a relatively long fragment of RNA has more “stems” than “loops,” then the usage of G2f3p should grow inside it. Therefore, we calculated the amount of nucleotides in “stems” (according to the RNAfold predictions) in fragments 400 nucleotides in length along the length of Zika RNA plus strand. Surprisingly, the correlation between the percent of nucleotides in “stems” and the usage of G2f3p has been found only in one long area of the RNA plus strand (from codon 600 until codon 1,730), as it is shown in Figure 3.

**Figure 4:**
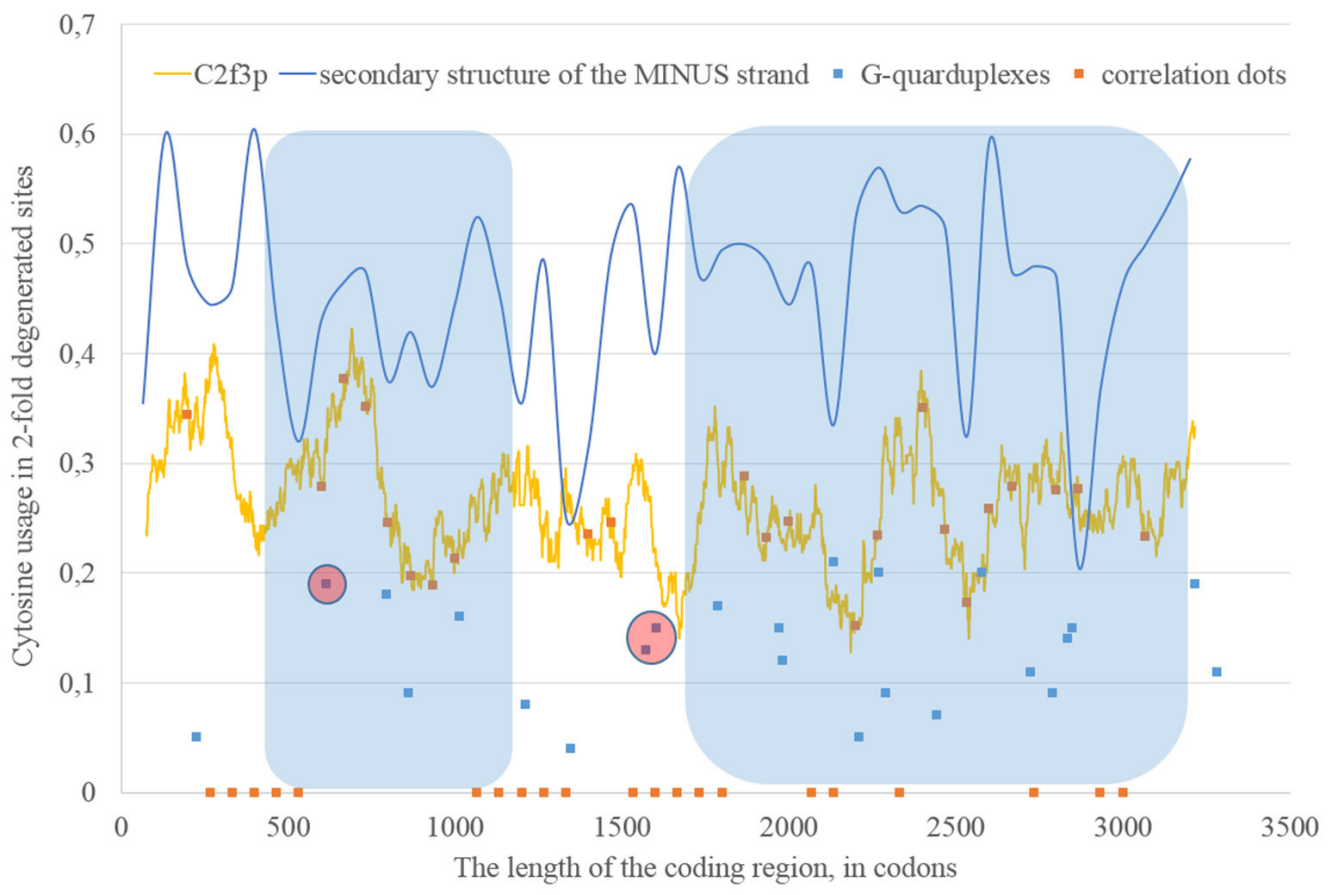
**The areas of correlation (blue areas) between the amount of nucleotides forming secondary structure of the RNA minus strand from ZIKV type 1 and the usage of cytosine in two-fold degenerated sites from third codon positions (C2f3p)**. Positions of predicted G-quarduplexes are shown. If correlation dots are situated on the C2f3p line, then the coefficient of correlation between C2f3p and the percent of stems in five windows (150 codons and 400 nucleotides in length, respectively) is higher than 0.3.

If we consider the possibility of ADAR editing of the RNA plus strand, then we should consider the possibility of ADAR editing of the RNA minus strand as well. In such case, the regions of RNA minus strand with high level of nucleotides forming “stems” should be also enriched by guanine. In other words, there should be correlation between the content of “stems” on the RNA minus strand and the usage of C2f3p on the RNA plus strand. As one can see in Figure 4, this correlation can be observed in two long areas: from codon 460 until codon 1,130; from codon 1,730 until codon 3,200.

According to our results, for some fragments of RNA their secondary structure (namely, the percent of nucleotides forming hydrogen bonds with each other) influences the nucleotide content in two-fold degenerated sites from third codon positions, but in other fragments there is no such dependence.

### The usage of rare codons along the consensus open reading frame of East African Zika strains

Codon usage usually demonstrates intriguing patterns of non-randomness. Some biases in codon usage can be directly explained by the mutational pressure theory. For example, in Zika virus one can expect high usage of codons ending with adenine, if those codons contain four-fold degenerated sites in their third positions. However, certain codons may demonstrate extremely low usage in a giving organism or a group of closely related organisms. It is thought that the level of tRNAs for such codons is low because of the low copy number of corresponding genes, their repression or problems with corresponding tRNA-amynoacyl-synthetazes (Wolin and Walter, 1988; Letzring et al., 2010). Alternative hypothesis stays that there may be repression of the usage of CpG and ApU dinucleotides causing the decrease of usages of codons containing such combinations (Tulloch et al., 2014). Anyway, the usage of certain codons decreases. After that, the system dealing with such rare codons begins to work slower (since there is no more strong negative selection keeping its velocity on a high level). Finally, rare codons become able to cause translation pausing (Rosenblum et al., 2013; Dana and Tuller, 2014). Ribosome stalls for a longer period of time when there are several rarely used codons situated near each other (Wolin and Walter, 1988). If such translational pause happens, the fragment of RNA in the 3′-direction from stalled ribosome becomes exposed to oxidative damage and RNA editing. Indeed, viral RNA plus strand should first be cleaned from proteins (Byk et al., 2016), then its secondary structure should be unwound by an enzyme with RNA-helicase activity (Jaramillo et al., 1991; Marintchev, 2013). If ribosome stalls somewhere in the middle of RNA, then the fragment in the 3′-direction will have enough time to form the secondary structure again and to be bound by ADAR. If it is so, then there should be a group of rarely used codons before the beginning of the area with the correlation between the percent of nucleotides in “stems” and G2f3p. However, in Figure 3 one can see that there are no such groups of rarely used codons in the beginning of the area, but the strongest “stop signal” for ribosome can be found in the middle of that area.

We showed the positions of groups of rarely used codons (in human and primates) in Figure 3. Indeed, in the middle of the abovementioned area there is a sequence represented below: **GUA**/**GUA**/GAC/CCU/AUU/GUG/**GUA**/GGA/**CUA**/CUG/**UUA**. There are five rarely used codons among 12. Therefore, there is a high probability that ribosome will be stalled in this sequence during translation of Zika RNA plus strand in the beginning of the infection (after the entrance of viral genome into the cytoplasm).

Therefore, there may be a kind of a local translation-associated mutational pressure in Zika virus. Probably, the area of correlation from the Figure 3 may be divided into two parts. The second part of this area really starts from the ribosome “stop signal.” There should be another mechanism responsible of the existence of the first part of the area of correlation.

### G-quarduplexes along the length of RNA strands for East African Zika strains

The fragment of viral RNA may become exposed to oxidative damage and RNA-editing enzymes in case of a pause in replication. One of the causes of RNA-dependent-RNA-polymerase stalling is the formation of G-quarduplexes by a single-stranded RNA (Cea et al., 2015). G-quarduplexes are formed by guanine-rich fragments of RNA. Positions and scores of G-quarduplexes have been predicted for Zika RNA plus strand (Figure 3) and RNA minus strand (Figure 4) with the help of the QGRS Mapper. The number of suspected regions is rather high for RNA plus strand, relatively to RNA minus strand. Interestingly, there are two regions that may form G-quarduplex near the beginning of the area with the correlation between the percent of nucleotides in “stems” on RNA plus strand and G2f3p (Figure 3). There are also such regions that may form G-quarduplexes before or soon after the start of both areas with the correlation between the percent of nucleotides in “stems” on RNA minus strand and C2f3p (Figure 4). Therefore, we cannot except the possibility of RNA-polymerase stalling before abovementioned areas. It is known that after the pause in replication viral RNA-dependent-RNA-polymerase continues the replication at significantly lower rate (Vilfan et al., 2008). Then the rate of replication may become normal again (Vilfan et al., 2008). It is also confirmed that one of the DNA-editing enzymes (Activation induced cytosine deaminase—AID) binds single-stranded fragments of DNA from immune cells (and introduces mutations in them) only in case if the rate of transcription is low enough to let it make its job (Canugovi et al., 2009). So, a “big wave” of G2f3p from Figure 3 might grow so high because that area is edited by ADAR during the translation (when the 3′ area after the sequence of rare codons is cleaved from capsid proteins) and during replication (when the 5′ area after the G-quarduplex is unwound). Several “low waves” of G2f3p from Figure 3 are not so high, probably, because they are affected by RNA-editing enzymes only during the polymerase stalling.

We can hypothesize about local replication-associated mutational pressure in Zika virus due to ADAR-editing of its plus and minus RNA strands during the pauses in replication. The hypotheses described above has been confirmed on consensus sequences of two other Zika lineages.

### The study of the consensus sequence of Zika type 2 (West African lineage)

**Figure 5:**
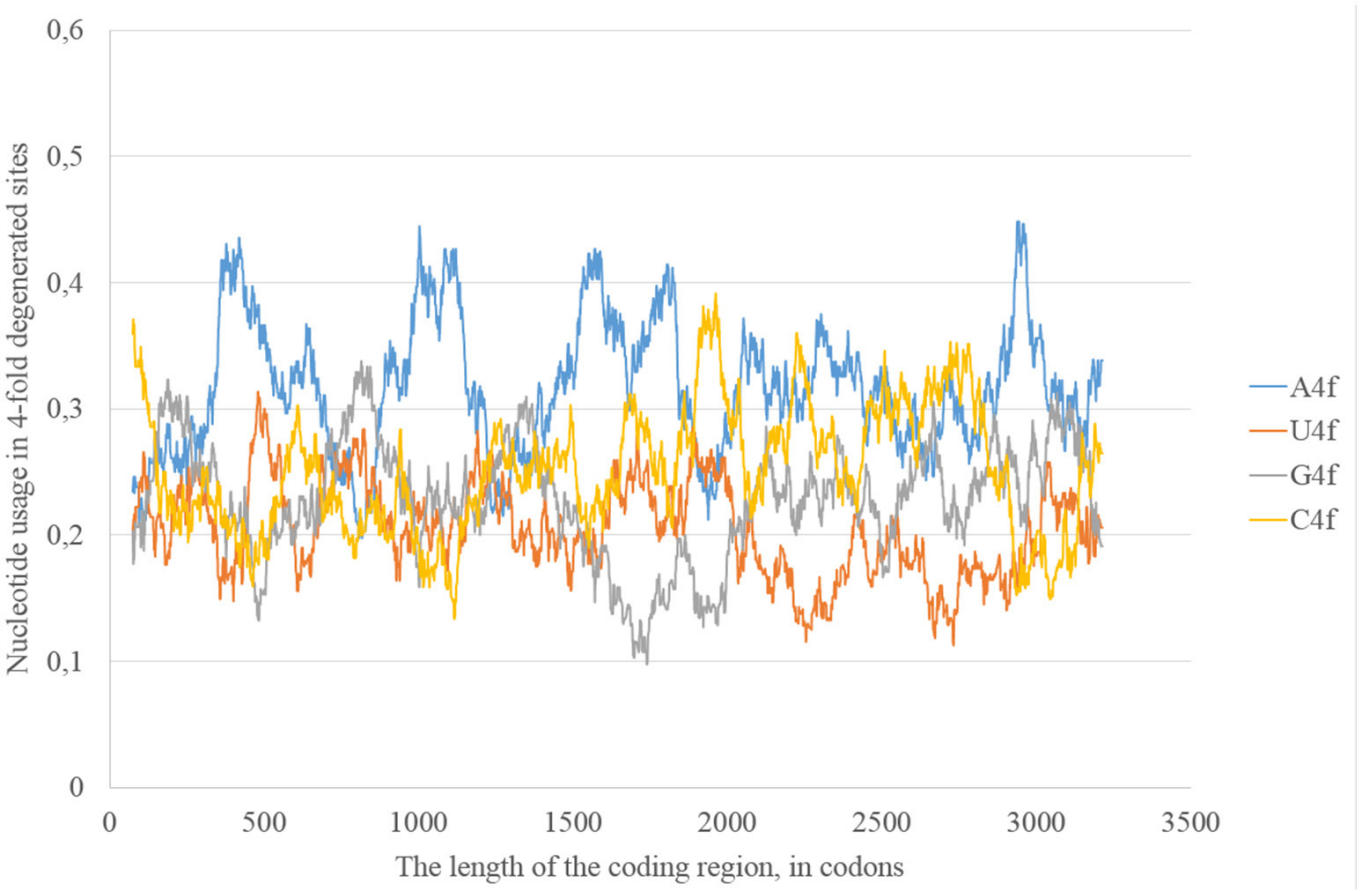
**Nucleotide usage in four-fold degenerated sites along the length of the consensus sequence for West African (type 2) ZIKV strains**.

In the consensus sequence of Zika type 2 cluster nucleotide usage biases are similar (in terms of overall nucleotide usages) to those from Zika type 1 consensus sequence, but rather different in positions and heights of some peaks (Figure 5). The distribution of “stems” is also different for two types of the same virus.

**Figure 6:**
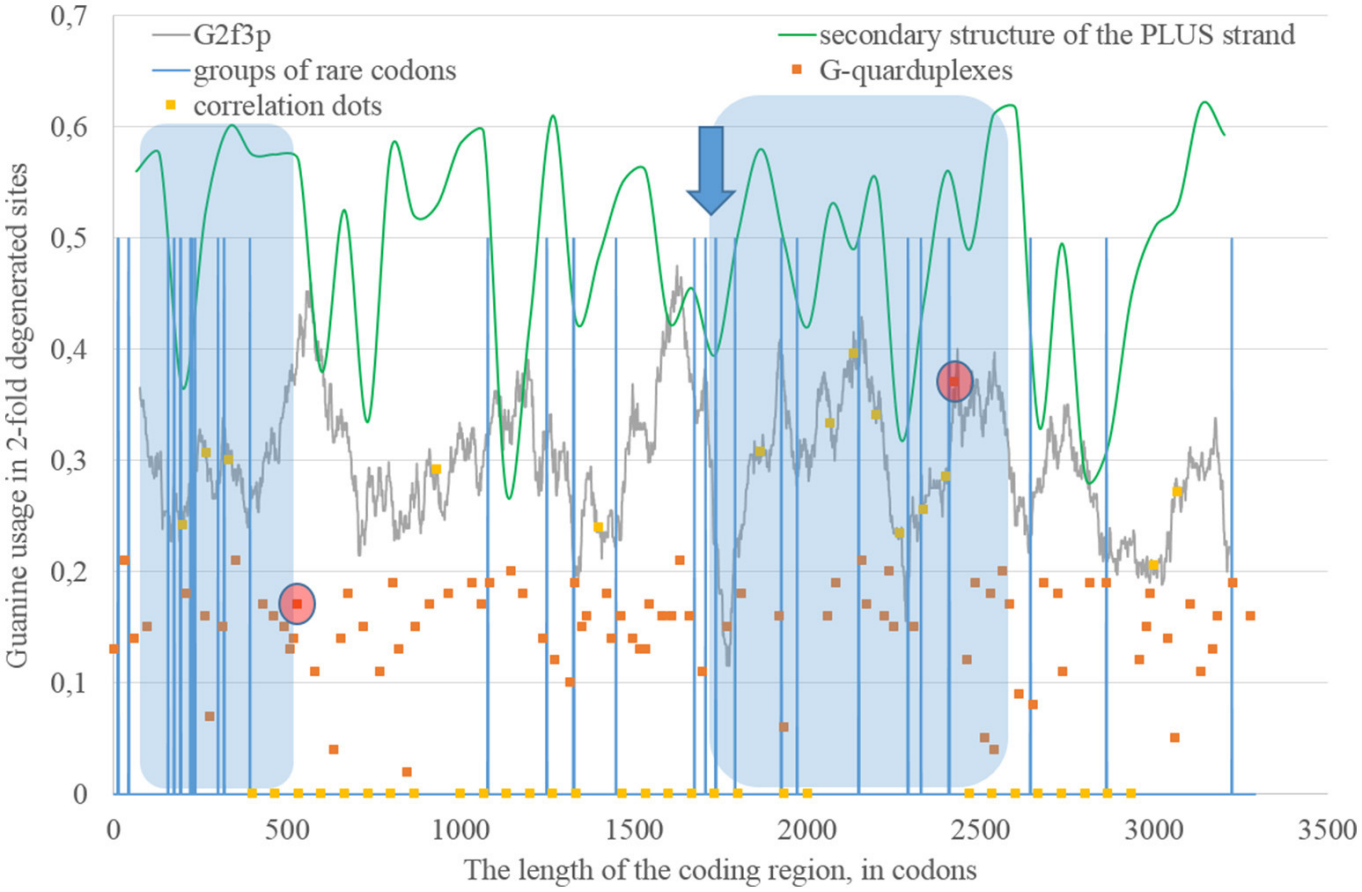
**The areas of correlation (blue areas) between the amount of nucleotides forming secondary structure of the RNA plus strand from ZIKV type 2 and the usage of guanine in two-fold degenerated sites from third codon positions (G2f3p)**. Positions of groups of rare codons and predicted G-quarduplexes are shown. If correlation dots are situated on the G2f3p line, then the coefficient of correlation between G2f3p and the percent of stems in five windows (150 codons and 400 nucleotides in length, respectively) is higher than 0.3.

Long areas with the correlation between the percentage of nucleotides forming “stems” on the RNA plus strand and the level of G2f3p are located between codons 80 and 460, 1,730, and 2,530 (Figure 6). There are numerous G-quarduplex sequences at the 5′-end of the first area and the group of rare codons inside it. There is the predicted G-quarduplex with the highest score situated near the 5′-end of the second area and there are two rare “CUA” codons going one after another before the beginning of that area (near its 3′-end). About 220 nucleotides downstream of the abovementioned G-quarduplex with the highest score there is another G-quarduplex sequence that was shown to be conserved in Flaviviruses (Fleming et al., 2016). It demonstrates the highest thermal stability *in vitro* among other sequences, which are able to form G-quarduplexes tested in that study (Fleming et al., 2016).

**Figure 7:**
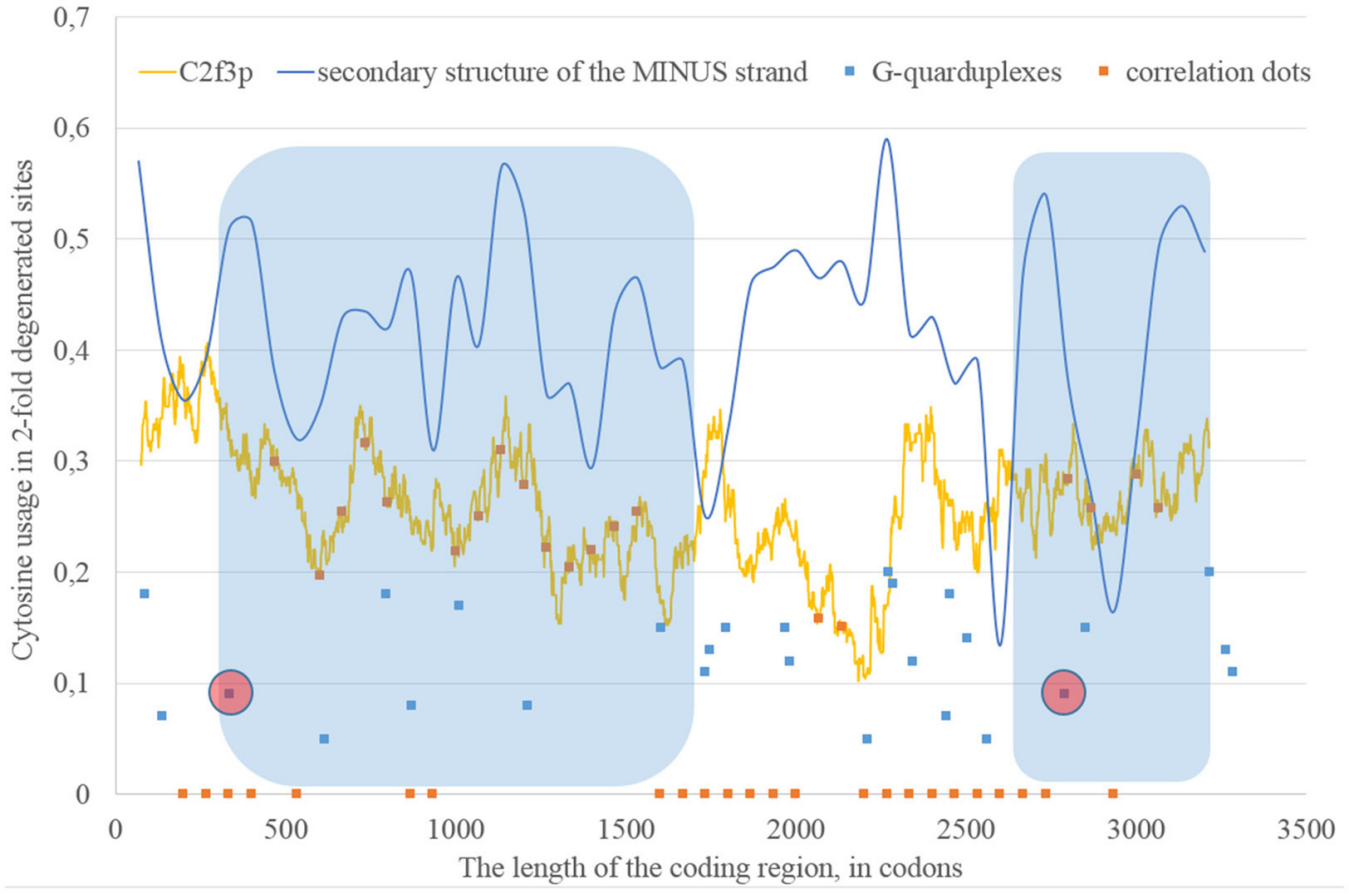
**The areas of correlation (blue areas) between the amount of nucleotides forming secondary structure of the RNA minus strand from ZIKV type 2 and the usage of cytosine in two-fold degenerated sites from third codon positions (C2f3p)**. Positions of predicted G-quarduplexes are shown. If correlation dots are situated on the C2f3p line, then the coefficient of correlation between C2f3p and the percent of stems in five windows (150 codons and 400 nucleotides in length, respectively) is higher than 0.3.

Long areas with the correlation between the percentage of nucleotides forming “stems” on the RNA minus strand and the level of C2f3p are located between codons 330 and 1,670, as well as between codons 2,670 and 3,200. There are predicted G-quarduplexes soon after the beginning of both abovementioned areas (Figure 7).

### The study of the consensus sequence of epidemic Zika type (Asian lineage)

**Figure 8:**
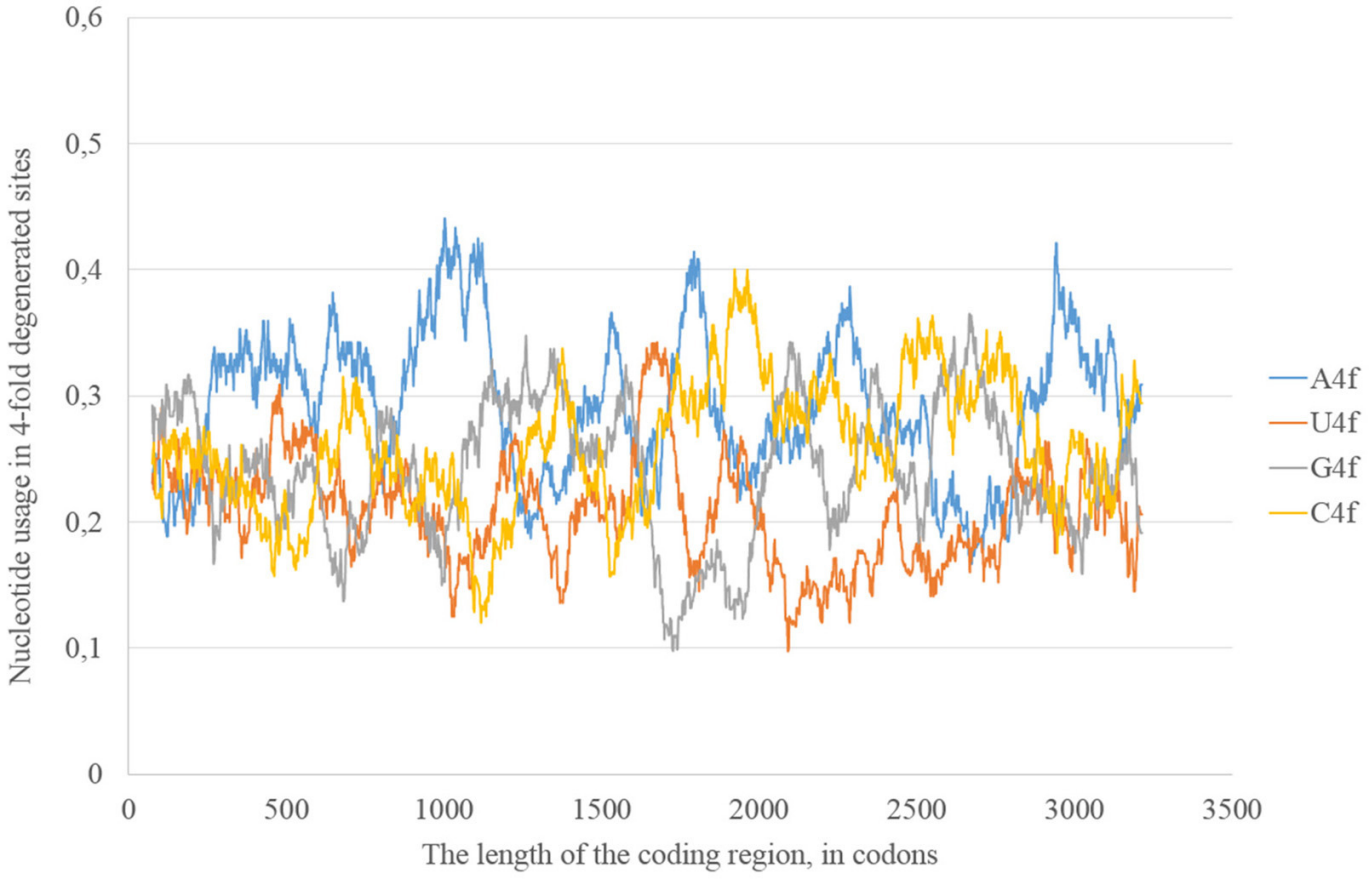
**Nucleotide usage in four-fold degenerated sites along the length of the consensus sequence for epidemic (Asian; type 3) ZIKV strains**.

**Figure 9:**
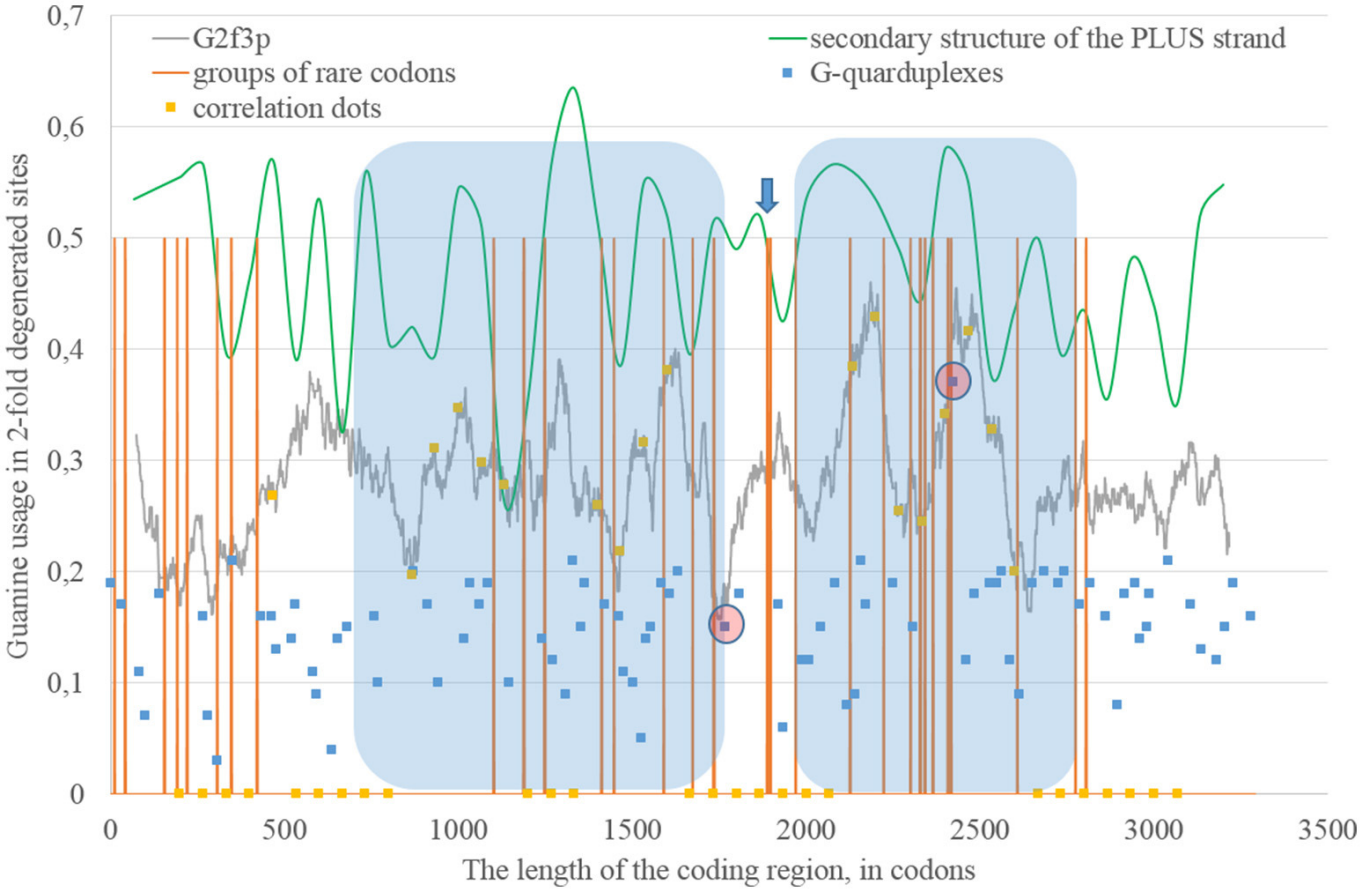
**The areas of correlation (blue areas) between the amount of nucleotides forming secondary structure of the RNA plus strand from ZIKV type 3 (epidemic) and the usage of guanine in two-fold degenerated sites from third codon positions (G2f3p)**. Positions of groups of rare codons and predicted G-quarduplexes are shown. If correlation dots are situated on the G2f3p line, then the coefficient of correlation between G2f3p and the percent of stems in five windows (150 codons and 400 nucleotides in length, respectively) is higher than 0.3.

The epidemic Zika type has some distinctive properties; however, the general direction of mutations in four-fold degenerated sites is the same as in other types of Zika: A4f demonstrates the highest peaks (Figure 8). There are two areas with the correlation of “stems” content and G2f3p on the RNA plus strand (Figure 9) from codon 730 until codon 1,730, from codon 2,000 until codon 2,670. There are no groups of rare codons before the first area, but there is a predicted G-quarduplex in its 3′-end (Figure 9). It means that the polymerase of epidemic Zika virus may be stalled on the RNA plus strand near the codon 1,730 and not the ribosome near the codon 730.

There is also the second area with the correlation between the percent of “stems” on the RNA plus strand and G2f3p (from codon 2,000 until codon 2,730) situated after the set of rare codons given below: **CGU**/GUC/**AUA**/GAU/UCC/AGG/AGA/UGC/**CUA**/AAG/**CCG**/GUC/**AUA**.

The most probable G-quarduplex can be found inside the second area from Figure 9, as well as the nearby G-quarduplex region that has already been studied *in vitro* (Fleming et al., 2016). Interestingly, the first area from the Figure 9 (putatively replication-associated one) demonstrates lower “waves” of G2f3p, while the second area (putatively both replication and translation-associated one) has higher “waves.” Similar situation has been described for Zika type 1 and type 2.

The number of “dots” with the correlation between the percent of “stems” on the RNA minus strand and the usage of C2f3p on the RNA plus strand is relatively low for the epidemic Zika virus. We can distinguish two areas of correlation: from codon 600 until codon 1,270 and from codon 1,460 until codon 2,530. The first area starts from the predicted G-quarduplex. There is no any G-quarduplex sequence in the beginning of the second area. Probably, there is just a residual correlation between C2f3p and the percent of nucleotides in “stems” of RNA minus strand in the first half of the first area, while the “stop signal” for polymerase is situated downstream.

The changes in positions of rare codons, in secondary structure and in positions of G-quarduplexes (together with other factors) cause mosaic distribution of changes in nucleotide usage if we compare consensus sequence of epidemic lineage with the one for East African lineage (**Figure 11**). In some areas the usage of adenine in two-fold degenerated sites is higher in the epidemic sequence relative to the reference one, in others the usage of guanine is higher (**Figure 11A**). The same situation can be seen with uracil and cytosine usages (**Figure 11B**). However, the overall tendency for all the types of Zika virus genomes is as follows: adenine is growing in four-fold degenerated sites, while guanine is growing in two-fold degenerated sites.

### Directions of mutational pressure along the length of Zika sequences

**Figure 10:**
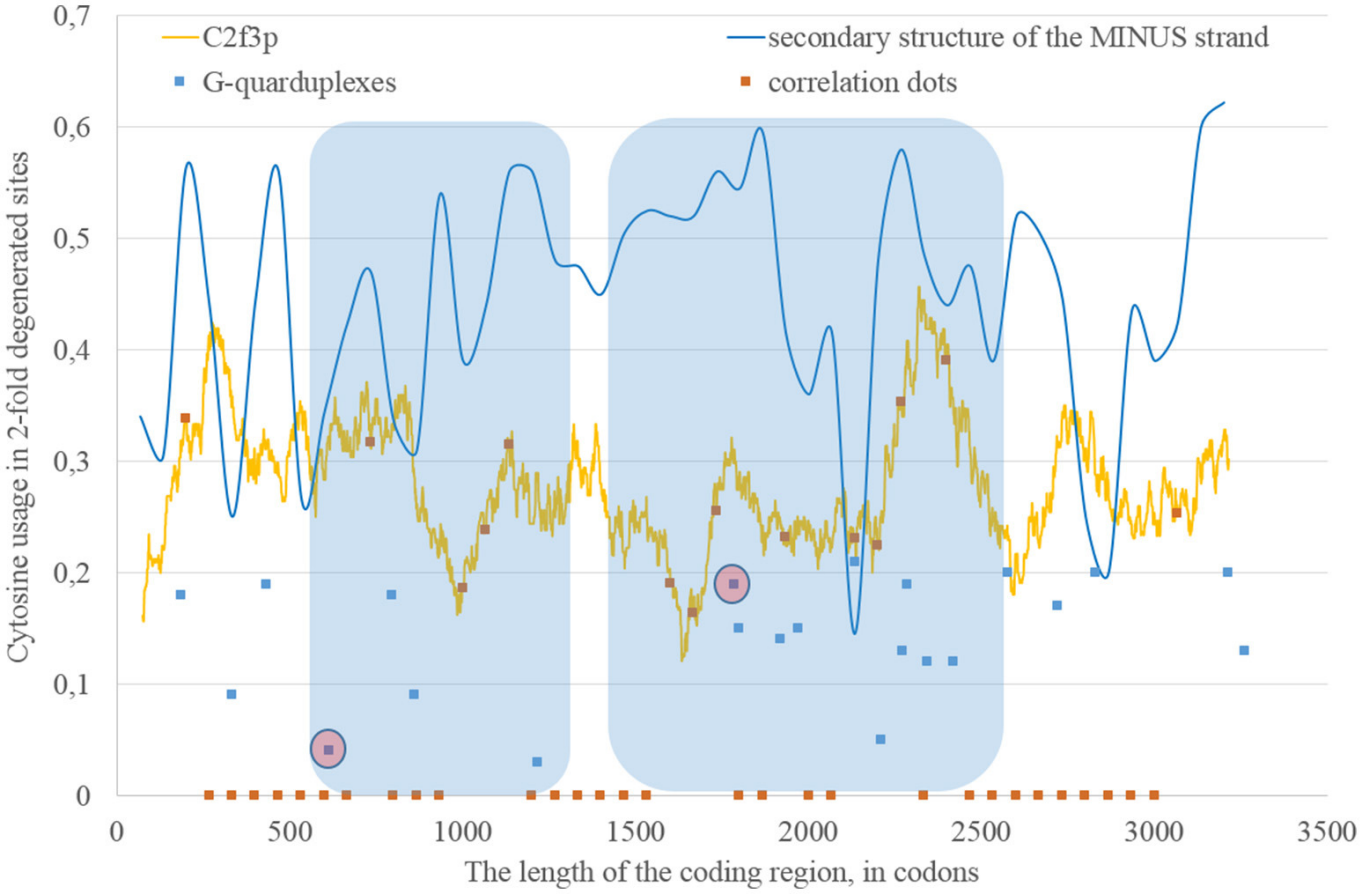
**The areas of correlation (blue areas) between the amount of nucleotides forming secondary structure of the RNA minus strand from ZIKV type 3 (epidemic) and the usage of cytosine in two-fold degenerated sites from third codon positions (C2f3p)**. Positions of predicted G-quarduplexes are shown. If correlation dots are situated on the C2f3p line, then the coefficient of correlation between C2f3p and the percent of stems in five windows (150 codons and 400 nucleotides in length, respectively) is higher than 0.3.

**Figure 11:**
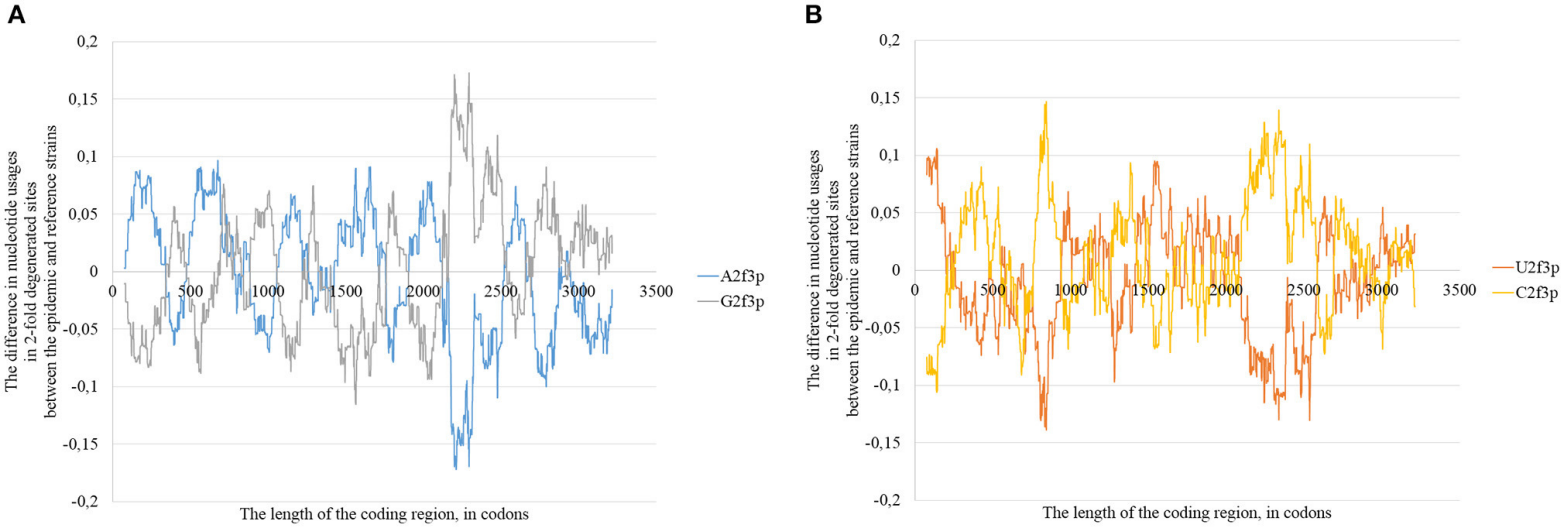
**The difference between the usages of A2f3p and G2f3p along the lengths of the consensus sequence for epidemic (type 3) ZIKV strains and type 1 ZIKV strains (A)**, as well as between the usages of U2f3p and C2f3p **(B)**.

Nucleotide usage biases are reflections of the mutational pressure, while that kind of reflection is similar to the starlight: we still see them after (sometimes) millions of years since the cause of those biases had disappeared. That is why it is necessary to estimate the direction of mutational pressure using the method taking into account nucleotide mutations in the whole alignment of sequences. The “VVTAK VarInvar” algorithm finds what types of nucleotides are less mutable than others by the way of comparison of their usages in invariable and in all stable sites. That method confirmed (Table 1) that throughout the most of the length of all three Zika types adenine and guanine usages are not decreasing in two-fold degenerated sites, while uracil and cytosine in two-fold degenerated sites are quite mutable. There are several deviations from the common tendency. The usage of uracil is growing only in one area from the epidemic Zika genome (from codon 200 until codon 800). Cytosine usage is growing in the region from codon 2,000 until codon 2,600 for the epidemic Zika type. Indeed, there is a peak of C2f3p usage in that region which is associated with the peak of the secondary structure content of the RNA minus strand (Figure 10). Interestingly, the usage of adenine is decreasing in the epidemic Zika virus in the nearby area (from codon 2,200 until codon 2,800), that can be observed in Figure 11A.

**Table 1:** **Directions of mutational pressure in two-fold degenerated sites from third codon positions along the length of type 1 (including reference sequence), type 2, and type 3 (including epidemic one) Zika genomes**.

**Zika type codons**	**A2f3p**			**U2f3p**			**G2f3p**			**C2f3p**		
	**1 (ref)**	**2**	**3 (epid)**	**1 (ref)**	**2**	**3 (epid)**	**1 (ref)**	**2**	**3 (epid)**	**1 (ref)**	**2**	**3 (epid)**
1–400	+	=	+	−	−	−	+	+	+	−	−	−
200–600	−	=	−	−	−	+	+	+	+	=	−	−
400–800	−	=	+	−	−	+	+	+	+	−	−	−
600–1000	+	=	+	−	=	=	+	=	+	−	−	−
800–1200	+	−	+	−	=	−	+	+	+	−	=	−
1000–1400	+	+	+	−	−	=	+	+	+	−	−	−
1200–1600	=	=	+	=	−	−	+	+	+	=	−	−
1400–1800	+	=	+	−	−	−	+	+	+	−	−	−
1600–2000	+	+	+	−	−	−	+	+	+	−	−	−
1800–2200	+	+	+	−	−	−	+	+	+	−	−	−
2000–2400	+	=	=	−	=	−	+	=	+	−	=	+
2200–2600	+	+	−	−	−	−	+	+	+	−	−	+
2400–2800	+	=	−	−	=	−	+	+	+	−	=	−
2600–3000	+	=	+	=	=	−	+	=	+	−	=	−
2800–3200	+	+	+	=	=	−	+	=	+	−	−	−

The increase of the usage of a given nucleotide is shown by “+” symbol, the decrease is shown by “−,” the equilibrium is shown by “=.”

The same kind of analysis for four-fold degenerated sites showed that adenine and guanine usages are growing due to the decrease of uracil and cytosine usages in all the three types of Zika (Table 2). However, there are some local deviations from this tendency. The largest deviation can be found in epidemic Zika virus (from codon 1,200 until codon 2,200), where cytosine usage is growing. This area (there are actually two peaks of C4f in that area in Figure 8) makes an overlap with the area of cytosine usage growth in two-fold degenerated sites.

**Table 2:** **Directions of mutational pressure in four-fold degenerated sites along the length of Zika type 1 (including reference sequence), type 2, and type 3 (including epidemic one) genomes**.

**Zika type codons**	**A4f**			**U4f**			**G4f**			**C4f**		
	**1 (ref)**	**2**	**3 (epid)**	**1 (ref)**	**2**	**3 (epid)**	**1 (ref)**	**2**	**3 (epid)**	**1 (ref)**	**2**	**3 (epid)**
1–400	+	+	+	−	−	−	+	+	+	−	−	−
200–600	+	+	+	−	=	−	+	+	+	−	−	−
400–800	+	+	+	−	=	−	+	=	+	−	−	−
600–1000	+	+	+	−	−	−	+	−	+	−	=	−
800–1200	+	+	+	−	−	−	−	+	+	−	=	−
1000–1400	+	+	=	−	−	−	=	+	+	−	=	−
1200–1600	+	+	−	−	−	−	=	+	+	−	−	+
1400–1800	+	+	+	−	−	−	+	+	−	−	−	+
1600–2000	+	+	+	−	−	−	+	+	+	−	−	+
1800–2200	+	+	=	=	−	−	=	+	+	−	−	+
2000–2400	+	+	+	−	−	−	−	=	+	−	=	−
2200–2600	+	+	+	=	−	−	=	=	+	−	=	−
2400–2800	+	+	+	−	−	−	=	+	+	−	=	−
2600–3000	+	=	+	−	=	−	=	=	+	=	=	−
2800–3200	+	+	+	−	=	−	+	=	+	−	=	+

The increase of the usage of a given nucleotide is shown by “+” symbol, the decrease is shown by “−,” the equilibrium is shown by “=.”

### Nucleotide content and secondary structure of RNA: what is the cause and what is the consequence?

It is known that the higher the usage of guanine, the higher the amount of secondary structure in the fragment of RNA. Guanine is able to form the most stable wobble base pair “G:U.” Because of this fact, computer algorithms consider “G:U” pairs when they predict the secondary structure of nucleic acids (Hofacker and Stadler, 2006). Taking this fact in consideration, one can suggest that the elevated usage of guanine is the cause, while the high percent of nucleotides in “stems” is the consequence. In this study we state that the situation is opposite: the percent of secondary structure is the cause, while the guanine usage is the consequence. To find the answer to this question for Zika virus we found the “dots” of correlation between the guanine usage and the percent of “stems” in RNA plus strand, as well as between the cytosine usage and the percent of “stems” in RNA minus strand, for three locations of guanine and cytosine residues. If the correlation is better in two-fold degenerated sites, than in four-fold degenerated sites and in all the sites occupied by guanine, then it is more likely that the secondary structure is the cause of mutational pressure. If the situation is opposite, then it is more likely that nucleotide usage is the cause of changes in secondary structure.

**Figure 12:**
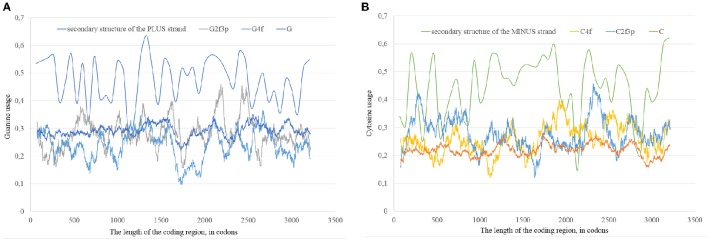
**The total usage of guanine (G), the usage of guanine in four-fold degenerated sites (G4f), the usage of guanine in two-fold degenerated sites from third codon positions (G2f3p), and the amount of secondary structure along the length of ZIKV type 3 consensus sequence (A)**. The total usage of cytosine (C), the usage of cytosine in four-fold degenerated sites (C4f), the usage of cytosine in two-fold degenerated sites from third codon positions (C2f3p), and the amount of secondary structure along the length of ZIKV type 3 consensus sequence **(B)**.

In our case (Table 3) for the RNA plus strand and the usage of guanine the answer is unclear. In Zika type 1 the percent of “dots” with the correlation is approximately the same if we calculate it for G2f3p, G4f and G. For Zika type 2 and type 3 the highest number of “dots” with correlation belongs to the total guanine usage. However, in Zika type 3 the difference between the percent of “dots” for G and G2f3p is lower. So, one may suggest that variations in total guanine usage should influence the amount of secondary structure on the RNA plus strand. This suggestion may be disproved by the data from Figure 12A where it is clearly seen that the variations in total usage of G are much narrower than variations in G4f and, especially, in G2f3p.

**Table 3:** **The percent of “dots” with the correlation between the amount of “stems” and the nucleotide usage for consensus sequences of three types of Zika**.

**Zika type**	**Type 1 (ref)**	**Type 2**	**Type 3 (epid)**
Plus strand/G	29.55	50.00	52.27
Plus strand/G4f	29.55	22.72	40.91
Plus strand/G2f3p	27.27	31.82	40.91
Minus strand/C	34.09	25.00	29.55
Minus strand/C4f	27.27	34.09	20.45
Minus strand/C2f3p	52.27	45.45	31.82

For the secondary structure of RNA minus strand and the usage of cytosine (in RNA plus strand) the tendency is clear. The highest percent of “dots” with correlation has been found for C2f3p and not for C4f or the total usage of C. It means that the percent of “stems” in RNA minus strand is the factor determining cytosine usage mostly in two-fold degenerated sites. Interestingly, the number of “dots” with such correlation for epidemic Zika (31.82%) is lower than those for two other types of Zika (52.27 and 45.45%). There is no even a correlation between the usages of C2f3p and C4f (*R* = −0.013) along the length of consensus sequence for epidemic Zika virus (Figure 12B).

In all three types of Zika virus the rates of C to T and T to C transitions are higher than the rates of G to A and A to G transitions. This fact is approved by the comparison of the ratio between variable sites containing T or C and A or G in two-fold degenerated sites with the ratio of all two-fold degenerated sites containing T or C and A or G in third positions. As one can see in Table 4, the real ratio between variable sites is always higher than the expected ratio. This fact is the evidence that RNA minus strand of Zika virus becomes a target for ADAR-editing more frequently than RNA plus strand.

**Table 4:** **Comparison between the real ratio of TC and AG variable sites in two-fold degenerated sites and their expected ratio along the length of Zika type 1 (including reference sequence), type 2, and type 3 (including epidemic one) genomes**.

**Zika type**	**Type 1 (Ref)**		**Type 2**		**Type 3 (Epid)**	
**Ratios codons**	**TC/AG Real**	**TC/AG Expected**	**TC/AG Real**	**TC/AG Expected**	**TC/AG Real**	**TC/AG Expected**
1–400	2.0	1.0	1.4	1.0	2.6	1.0
200–600	1.5	1.1	1.6	1.0	1.8	1.1
400–800	1.5	1.2	2.1	1.1	1.3	1.0
600–1000	2.6	1.1	1.3	1.1	1.5	1.0
800–1200	2.1	0.9	0.9	0.8	2.0	0.9
1000–1400	2.0	0.9	2.0	0.8	1.6	0.9
1200–1600	1.7	1.0	3.0	1.0	2.7	1.0
1400–1800	2.0	1.0	2.8	0.9	3.1	1.0
1600–2000	2.1	0.9	3.5	0.9	2.9	0.9
1800–2200	1.3	0.8	3.0	0.8	2.6	0.8
2000–2400	1.1	0.9	1.0	0.8	0.9	0.9
2200–2600	2.0	0.8	1.8	0.8	0.9	0.8
2400–2800	1.7	0.9	1.2	0.9	1.1	0.9
2600–3000	1.2	0.8	0.9	0.8	1.8	0.8
2800–3200	1.4	0.8	1.1	0.8	1.7	0.7

### Mutations in ADAR2-specific trinucleotide motifs

It is known that ADAR2 enzyme prefers to deaminate adenines situated in four trinucleotide motifs: UAG; UAU; AAG; and AAU (Lehmann and Bass, 2000). The overall percentage of adenine residues that mutated at least in a single sequence from the East Africa lineage of Zika (relative to the consensus sequence) is equal to 6.32%. The percentage of mutated adenines in UAU, AAG, and AAU trinucleotides is approximately the same as an overall percentage (the differences are insignificant). In contrast, the percentage of mutated adenines in UAG motif (29.03%) is significantly higher than the overall percentage (*P* < 0.05). In the RNA minus strand of the East Africa Zika lineage adenine residues in UAG motif mutated in 21.34% of cases, adenine residues in UAU motifs mutated in 23.08% of cases, while the overall percentage of mutated adenine residues is equal to 11.10%. These data give additional confirmation of the ADAR editing of Zika virus genome.

The same test has been applied to West Africa lineage of Zika. On the RNA plus strand the percentage of UAG motifs with mutated adenine is equal to 16.30%. This percentage is significantly higher than the overall percentage of mutated adenines (4.14%). However, the percentage of AAU motifs with mutated adenine residues (1.67%) is significantly lower than the overall percentage of mutated adenines. On the RNA minus strand percentages of UAG (12.82%) and UAU (13.68%) motifs with mutated adenine residues are higher than the overall percentage of mutated adenines (7.30%), but the difference is not significant.

In the RNA plus strand of epidemic Asian Zika lineage the percentage of UAG motifs with mutated adenine is significantly higher than the overall percentage of mutated adenine (18.18 vs. 6.28%, *P* < 0.05). However, the percentage of UAU motifs with mutated adenines (2.60%) is significantly lower than the overall percentage. In the RNA minus strand of epidemic Zika lineage the percentage of UAG motifs with mutated adenine residues is significantly higher than the overall percentage of mutated adenines (24.14 vs. 10.76%, *P* < 0.05).

In general, the rate of adenine mutations is elevated in one of the four ADAR2 specific trinucleotide motifs (UAG). In some cases described above the rate of adenine mutations may be lower in such motifs as UAU and AAU. In our opinion, such motifs are completely GC-poor, and so the probability that they will form secondary structure is low. First of all, to be edited by ADAR each motif must be included in the double stranded region. If overall GC-content of a sequence is high, trinucleotides like UAU and AAU have a higher chance to be included in a double stranded region, and so to be edited by ADAR. If a sequence has average or low GC-content, then UAG trinucleotide has the highest probability to form secondary structure and to let ADAR2 to edit adenines in its preferable motif.

## Discussion

The implications of Codon usage bias (CUB) can be explained by mutational pressure and translational selection (Zhao et al., 2015). CUB can be influenced by definite factors and show marked consequences in different organisms (Guo et al., 2012). Synonymous codon usage can reveal the evidences of evolution for individual genes. Compositional constraints and translational selection are thought to play a major role in accounting for nucleotide usage variations in different organisms, but for some bacterial species it has already been approved that the CUB is primarily affected by strand-specific mutational pressure (Guo and Yuan, 2009). On one hand, CUB may be adapted to the levels of tRNAs in the given organism, on the other hand, levels of tRNAs may also be adapted to the existing CUB. These two processes should slowly work together in case if the accuracy and velocity of translation plays a significant role in the survival of a given species. One may expect that at a constant mutational pressure CUB of genes (especially highly expressed ones) will finally become adapted to the requirements of translation machinery (and vice versa). However, a sudden change in the mutational pressure direction may cause a disadaptation of CUB and the levels of corresponding tRNAs. Especially interesting situation exists when there are different CUBs along the length of the same coding region. According to our data, the number of groups of rare codons for epidemic ZIKV strain is less than that for type 1 ZIKV (50 vs. 56), but it is higher than that for the type 2 ZIKV (47). So, we cannot state that the epidemic ZIKV has the most adopted CUB to the translation system of primates.

Probably, there is also a kind of positive natural selection fixing those variants of viruses which have the less number of G-quarduplex regions. Indeed, the lower the number of G-quarduplexes, the higher the velocity and accuracy of replication (Stanton et al., 2016). Currently it is thought that eukaryotic organisms developed special mechanisms to prevent formation of G-quarduplexes both in DNA (Lehmann and Bass, 2000) and RNAs (Stanton et al., 2016), while prokaryotic organisms seem to avoid the usage of DNA fragments prone to form G-quarduplexes (Lehmann and Bass, 2000). It is hard to suggest that the eukaryotic machinery for G-quarduplexes unfolding can work well with viral RNA in the period of acute infection. So, probably, viruses follow a strategy of prokaryotic organisms and avoid the usage of sequences that cause pauses in replication. According to our results, RNA minus strand of epidemic ZIKV really has just 22 possible G-quarduplex regions, while type 1 ZIKV has 24 such regions, and type 2 ZIKV has 27. The decrease of the number of G-quarduplexes in the progenitor of epidemic ZIKV strains might result in the faster replication of daughter RNA plus strands and to the decrease of the length of regions prone to ADAR-editing.

Usually the strand of RNA that has a longer period of life in cells collects more nucleotide mutations and inherits them to the progeny (Khrustalev and Barkovsky, 2011). Many viruses are known to have more purine nucleotides (adenine and guanine) in their mRNAs than pyrimidine nucleotides (uracil and cytosine; Cristillo et al., 2001). Zika virus is not an exception from this rule (van Hemert and Berkhout, 2016). In ZIKV, RNA minus strand should play the role of the collector of mutations. The usage of uracil and cytosine is decreasing in ZIKV genome. However, there are certain areas of genome in which cytosine usage is growing. In comparison to reference ZIKV strain, the epidemic strain has remarkable difference in the distribution of cytosine usage in two-fold degenerated sites along the RNA plus strand. This corresponds to the distribution of guanine usage in minus strand of RNA. There are regions in which the amount of secondary structure dictates the usage of guanine in sites synonymous for A–G transitions. Existence of these regions can be explained for ADAR-editing of RNA minus strands during pauses in replication caused by polymerase stalling on G-quarduplex regions.

Even though there is a significant bias in nucleotide usages, the sum of guanine and cytosine usages (G+C) is close to 50% in Zika and other Flaviviruses (Jenkins et al., 2001). In general, adenine and guanine usages are growing in Zika genome. Adenine is more stable in four-fold degenerated sites, while guanine is more stable in two-fold degenerated sites from third codon positions. Certain local peaks of guanine usage in two-fold degenerated sites are linked to the areas with high amount of secondary structure on the RNA plus strand. Possible reasons behind are the ADAR-editing of RNA plus strand during pauses in translation caused by ribosome stalling on groups of rare codons and pauses in replication caused by polymerase stalling on G-quarduplex regions.

The number of regions of RNA minus strand demonstrating traces of ADAR-editing is lower for epidemic Zika sequences than for other types of the same virus. Probably, the number of points at which polymerase makes pauses on the RNA minus strand (including G-quarduplexes) is lower for epidemic type of Zika. This feature can be the cause of more accurate and fast replication of the viral strain in recent outbreaks of the decade.

## Conclusions

In this article, the mutational pressure direction of reference and epidemic ZIKV lineages has been estimated using nucleotide usage biases comparison and the analysis of nucleotide content in invariant and variable sites. The difference in nucleotide usage of three lineages has shown that genomic variations may be linked with the increased virulence of epidemic lineage by the mechanism of the decrease of number of points in which RNA-polymerase can be stalled during replication. The results have shown that guanine usage is growing in ZIKV RNA plus strand due to adenine to guanine transitions, while the nucleotide usage of adenine in four-fold degenerated sites prevails in ZIKV genome due to cytosine to adenine transversions. In certain areas of RNA plus strand of both reference strain and epidemic strain the usage of cytosine in two-fold degenerated sites shows direct dependence on the amount of secondary structure in minus strand RNA. There are also certain areas with the correlation between guanine usage in two-fold degenerated sites and the amount of secondary structure in plus strand. The presence of high amount of secondary structure and conserved G-quadruplexes in genomic RNA has resulted in increased ADAR-editing of RNA plus and RNA minus strands of ZIKV. These variations arisen due to areas associated with ADAR-editing and may resulted into the origin of epidemic strains. The lower amount of areas associated with ADAR-editing in the RNA minus strand of Asian ZIKV lineage could be the major cause behind the rise in the number of outbreaks in past decade.

## Author contributions

RG and VVK: conception and design, review of the manuscript and study supervision. VVK, TAK: acquisition of data, analysis and interpretation of data. VVK, TAK, NS, and RG: writing of the manuscript.

## Funding

This work has been funded by DST grant, India (YSS/2015/000613) that belongs to RG and Indian Institute of Technology Mandi.

### Conflict of interest statement

The authors declare that the research was conducted in the absence of any commercial or financial relationships that could be construed as a potential conflict of interest.
